# Genome Sequence of *Lactobacillus fermentum* Strain MTCC 8711, a Probiotic Bacterium Isolated from Yogurt

**DOI:** 10.1128/genomeA.00770-13

**Published:** 2013-09-26

**Authors:** Sathyanarayanan Jayashree, Sharma Pooja, Muthuirulan Pushpanathan, Udayakumar Vishnu, Jagadesan Sankarasubramanian, Jeyaprakash Rajendhran, Paramasamy Gunasekaran

**Affiliations:** Department of Genetics, Centre for Excellence in Genomic Sciences, School of Biological Sciences, Madurai Kamaraj University, Madurai, India

## Abstract

*Lactobacillus fermentum* strain MTCC 8711 is a lactic acid bacterium isolated from yogurt. Here, we describe the draft genome sequence and annotation of this strain. The 2,566,297-bp-long genome consisted of a single chromosome and seven plasmids. The genome contains 2,609 protein-coding and 74 RNA genes.

## GENOME ANNOUNCEMENT

*Lactobacillus fermentum* is a heterofermentative lactic acid bacterium (LAB) that belongs to the family *Lactobacillaceae* in the class *Bacilli* of the phylum *Firmicutes*. The LAB are generally recognized as safe (GRAS) and widely used in the production of fermented foods. *L. fermentum* is also considered a potential probiotic organism as it possess desirable probiotic properties such as nonpathogenicity to human, increased resistance to the environment prevailing in the intestine, and beneficial effects to the human immune system (1). *L. fermentum* strain MTCC 8711 was isolated from Vellore, Tamil Nadu, India. It is a riboflavin-producing strain and possesses several probiotic properties (2).

Using a DNeasy genomic DNA isolation kit (Qiagen), we isolated genomic DNA from a culture grown overnight. Whole-genome sequencing was performed using an Ion Torrent personal genome machine (Life Technologies, Carlsbad, CA). Sequencing was performed using 200-bp chemistry on an Ion 316 semiconductor chip. A total of 1,849,723 reads with an average read length of 115 bp were obtained, which yielded 213.1 Mb sequenced bases (~85-fold coverage of an ~2.5-Mb genome). Reference-based assembly was performed using MIRA (mimicking intelligent read assembly) version 3.9.17 with *L. ferrmentum* IFO 3956 (NC_010610) as the reference genome (3). A total of 109 contigs were obtained and the total consensus was 2,566,297 bp with a 49.7% GC content. The genome consisted of a single chromosome of 2,268,443 bp and seven plasmids. The plasmids were designated pLF01 to pLF07 with sizes of 53.03 kb, 64.51 kb, 36.60 kb, 14.06 kb, 31.56 kb, 46.59 kb, and 51.48 kb, respectively. The genome sequence was annotated using the RAST (Rapid Annotations using Subsystems Technology) server (4) and the NCBI Prokaryotic Genome Annotation Pipeline (http://www.ncbi.nlm.nih.gov/genomes/static/Pipeline.html). The 2,566,297-bp-long genome contains 2,609 protein-coding and 74 RNA genes.

The genome possesses genes responsible for the production of β-galactosidase and α-galactosidase, which play major roles in the conversion of β-galactosides of milk into simple sugars that help in the consumption of milk by lactose-intolerant people. Two genes coding for bile salt hydrolase (penicillin V acylase and choloylglycine hydrolase) were identified which could be responsible for the bile salt tolerance of this bacterium. Bile salt hydrolase helps in lowering cholesterol levels in humans. The genome contains a colicin V synthesis protein in the chromosome. In addition, two genes coding for holin proteins, which act as bacteriocins, were identified in two plasmids (pLF02 and pLF06). Bacteriocins improve the survivability of the organism in a competitive microbial environment like the human gut. According to the EU-PROSAFE project, it is recommended that probiotic strains should not contain transferable antibiotic resistance genes (5). Interestingly, though the genome of *L. fermentum* MTCC 8711 contains seven plasmids of different sizes, none of them have antibiotic resistance genes. Therefore, *L. fermentum* MTCC 8711 can be used as a probiotic strain for improving intestinal health.

### Nucleotide sequence accession number.

This whole-genome shotgun project has been deposited at DDBJ/EMBL/GenBank under the accession no. AVAB00000000.
